# Altered serum interleukin-17A and interleukin-23A levels may be associated with the pathophysiology and development of generalized anxiety disorder

**DOI:** 10.1038/s41598-024-66131-9

**Published:** 2024-07-02

**Authors:** Mamun-or-Rashid, A. S. M. Roknuzzaman, Rapty Sarker, Jannatul Nayem, Mohiuddin Ahmed Bhuiyan, Md. Rabiul Islam, Zobaer Al Mahmud

**Affiliations:** 1 Department of Clinical Pharmacy and Pharmacology, Faculty of Pharmacy, University of Dhaka, Dhaka, 1000 Bangladesh; 2 Department of Pharmacy, University of Asia Pacific, Dhaka, 1205 Bangladesh; 3 School of Pharmacy, BRAC University, Kha 224 Bir Uttam Rafiqul Islam Avenue, Progati Sarani, Merul Badda, Dhaka, 1212 Bangladesh

**Keywords:** Generalized anxiety disorder, Interleukin-17A, Interleukin-23A, Cytokines, Pathophysiology, Biomarker, Risk assessment, Cytokines, Biomarkers, Neurology

## Abstract

In recent times, the pathogenesis of generalized anxiety disorder (GAD) and the influence of pro- and anti-inflammatory cytokines on it have garnered considerable interest. Cytokine research, especially Th-17 cytokine research on GAD patients, is limited. Here, we aim to assess the role of interleukin-17A (IL-17A) and interleukin-23A (IL-23A) in the pathophysiology and development of GAD. This investigation included 50 GAD patients and 38 age-sex-matched healthy controls (HCs). A psychiatrist diagnosed patients with GAD and assessed symptom severity using the DSM-5 and the GAD-7 scales. The serum concentrations of IL-17A and IL-23A were determined using commercially available ELISA kits. GAD patients exhibited elevated levels of IL-17A (77.14 ± 58.30 pg/ml) and IL-23A (644.90 ± 296.70 pg/ml) compared to HCs (43.50 ± 25.54 pg/ml and 334.40 ± 176.0 pg/ml). We observed a positive correlation between disease severity and cytokine changes (IL-23A: r = 0.359, p = 0.039; IL-17A: r = 0.397, p = 0.032). These findings indicate that IL-17A and IL-23A may be associated with the pathophysiology of GAD. ROC analysis revealed moderately higher AUC values (IL-23A: 0.824 and IL-17A: 0.710), demonstrating their potential to discriminate between patients and HCs. Also, the sensitivity values of both cytokines were relatively higher (IL-23A: 80.49% and IL-17A: 77.27%). According to the present findings, there may be an association between peripheral serum levels of IL-17A and IL-23A and the pathophysiology and development of GAD. These altered serum IL-17A and IL-23A levels may play a role in directing the early risk of developing GAD. We recommend further research to ascertain their exact role in the pathophysiology and their performance as risk assessment markers of GAD.

## Introduction

Generalized anxiety disorder (GAD) is a persistent psychiatric disorder marked by ongoing and unmanageable levels of intense anxiety and worry, as well as excessive concerns about various aspects of life or activities^1,2^. It is a highly prevalent neuropsychiatric disorder affecting the daily, professional, family, and social lives of affected people and is thus considered one of the significant public health concerns^3^. At present, 1–6% of people worldwide are affected by GAD, and if it remains untreated, it can cause mental and cognitive disability, affecting the quality of life^2,4^. The available pharmacotherapies for GAD treatment include selective serotonin reuptake inhibitors (SSRIs), serotonin and norepinephrine reuptake inhibitors (SNRIs), and a high voltage-activated calcium channel blocker, pregabalin as first-line treatment options and benzodiazepines including diazepam as second-line therapy^4,5^. Besides these pharmacotherapies, some non-pharmacological interventions, such as cognitive behavioral therapy (CBT), are recommended for the management of GAD symptoms^6^.

GAD has multiple etiological factors, such as neurobiological, neurochemical, genetic, environmental, psychological, and immunological factors^4^. Though dysregulation in the monoaminergic pathway in CNS implicates in the pathogenesis of GAD, currently available drugs (e.g., SSRIs and anti-depressants) are ineffective in 42% of GAD cases^7^ or failed to display adequate efficacy in almost 50% of GAD patients^6^. It implies that the pathophysiology of GAD is poorly defined, and the available dominant hypothesis of dysregulated monoaminergic neurotransmission failed to explain the etiopathologies of GAD. Thus, further research is warranted to investigate the mechanisms underlying the disease progression in the case of GAD. The challenge in GAD management is finding an effective diagnostic and prognostic biomarker for accurate and early detection because such effective biomarkers for GAD are currently unavailable.

Neuroimaging techniques such as magnetic resonance imaging (MRI), diffusion tensor imaging (DTI), and positron emission tomography (PET) still have high value in clinical neuroscience research. Clinicians cannot use these techniques in their everyday clinical practice to detect GAD because these methods of diagnosis and prognosis are expensive, time-consuming, require expertise to conduct, and only restricted to specialized clinics. As therapeutic drug monitoring or disease progression requires multiple screening processes, these neuroimaging markers are not suitable as prognostic tools due to the high-cost burden. Currently, GAD is diagnosed based on the patient’s self-reported symptoms and the patient’s responses to the structured 7-item questionnaire. This subjective assessment is one of the causes of misdiagnosis or underdiagnosis of GAD that fails in therapeutic management or sub-optimal treatment intervention^8^. Accordingly, the lack of an objective diagnostic tool/biomarker ultimately increases the prevalence of GAD along with enhanced treatment or management costs. In this background, a blood-based effective biomarker for GAD is essential as it is less invasive, inexpensive, and easy to perform. In this regard, we hypothesize that pro-inflammatory cytokines have the potential to serve as blood-based diagnostic biomarkers for GAD and thus could serve to predict and diagnose the early stage of the disease and monitor therapeutic responses to drugs.

Research indicates that neuroinflammation, i.e., inflammatory responses in CNS are implicated in the pathogenesis or development of several neuropsychiatric disorders such as Alzheimer’s disease, Parkinson’s disease, major depressive disorder, panic disorder, bipolar disorder, obsessive–compulsive disorder, schizophrenia, etc.^9–13^. Several studies demonstrated that peripheral pro-inflammatory cytokines infiltrated into CNS can trigger excessive microglial or astrocytic activation that influence further release of inflammatory mediators such as IL-1β, IL-6, TNF-α, etc. These ultimately trigger a cascade of inflammatory reactions leading to excessive damage in neurocircuits related to serotonergic, dopaminergic, and adrenergic pathways associated with mood, behavior, and cognition. In addition, pro-inflammatory cytokines can alter the neurotransmitter systems by either diminishing monoamine (serotonin, dopamine, norepinephrine) production in CNS or can stimulate the glutamate-mediated excitotoxicity by enhancing oxidative bursts that ultimately result in excessive damage in neurocircuits associated with mood, depression, or anxiety states^1,10,11^.

Interleukin-17A (IL-17A), a member of the IL-17 family, is a pro-inflammatory cytokine that plays a vital role in mediating immune response and inflammation. IL-17A is secreted from Th17 cells and can trigger tissue injury and peripheral/central inflammation by upregulating the local inflammatory mediators such as IL-1β, IL-6, IL-8, and TNF-α^14–16^. It can also initiate the synthesis of extracellular matrix metalloproteinase and reactive oxygen or nitrogen species, resulting in tissue injury or the presence of autoimmune illnesses or neuro-inflammatory conditions^14,16^. IL-23 is a heterodimeric protein that consists of two subunits: IL-23A, or IL-23p19, and the p40 subunit of IL-12. IL-23A is released by several antigen-presenting cells including macrophages, dendritic cells, and B cells, in peripheral tissues. In the CNS, it is secreted by microglia, astrocytes, and macrophages that have transmigrated from the periphery to the CNS^17^. The primary role of IL-23A is to induce the differentiation and activation of Th17 cells. IL-23A attaches to IL-23A receptors on Th17 cells, triggering the activation of the STAT4 signaling pathway to release several pro-inflammatory mediators, such as IL-17, IL-22, TNF-α, and GM-CSF, from Th17 cells. The overstimulation of the IL-23A/IL-23R pathway leads to neuroinflammation in the central nervous system and autoimmune diseases in peripheral tissues^18^. Multiple preclinical and clinical investigations have shown that IL-23A plays a significant role in advancing and forming neuro-inflammation, which is involved in the pathophysiology of various neurodegenerative illnesses such as Alzheimer’s disease and neuropsychiatric disorders. Recent studies indicate correlations between inflammatory reactions and neuropsychiatric illnesses such as schizophrenia, major depressive disorder (MDD), and GAD^1,3,19,20^. Malfunctioning immunological responses, namely persistent mild inflammation, are associated with depression, and the use of the same medications for GAD and MDD indicates shared underlying causes^21,22^. However, there is a scarcity of research on the impact of dysregulated inflammatory responses in GAD.

Initial observations linked stress-induced anxiety to elevated levels of pro-inflammatory cytokines^23^. Several studies have identified connections between markers that promote inflammation and the degree of severity of GAD^1,19,24–29^. However, there is disagreement since some investigations have found no significant links between pro-inflammatory cytokine serum levels and GAD pathogenesis^2,3,30,31^. The IL-23A or IL-17A immunological axis, which is involved in autoimmune and inflammation-related diseases, might also have a function in the development of depression and anxiety disorders^16,32,33^. Several studies have linked the IL-17A and IL-23A pathways to anxiety and depression in both human subjects and animal models^33–38^. Preclinical research on mice indicates that IL-17A significantly contributes to the development of anxiety associated with epilepsy, stroke, or chronic stress^39–41^. Consistent with these findings obtained from animal model studies, IL-17A serum levels were found to be positively and independently correlated with the anxiety severity in rheumatoid arthritis patients even after adjustment for disease activity for arthritis and pain^42^. Similarly, IL-23A serum levels were reported to be significantly and positively correlated with the anxiety scores in psoriatic arthritis patients^43^. Although, several studies implicated the role of Th1 cytokine (IL-1β, IL-2, IL-12, TNF-α, and IFN-γ) and Th2 cytokines (IL-4, IL-6, IL-10) in the pathophysiology of different anxiety and depressive disorders^1,44^, only a very few studies were performed investigating the role of Th17 cytokines (IL-17, IL-22, IL-21) and Th17 cytokine stimulant, IL-23 in the pathogenesis of GAD. Only two case–control studies identified increased levels of Th17 cytokines including IL-17A in individuals with GAD^16,45^. However, they did not examine the association between IL-17A levels and the degree of anxiety severity. Furthermore, no research has been conducted to investigate the involvement of the IL-23A/IL-17A immune axis in GAD among patients from Bangladesh. Based on these backgrounds, the current study aimed to evaluate the role of IL-17A and IL-23A in the pathophysiology and development of GAD. The study also aimed to assess the predictive value of these two cytokines as risk indicators for the development of GAD.

## Methods

### Study design and participants

The present study recruited 50 GAD patients aged between 18 and 60 and 38 age, sex, and BMI-matched healthy controls (HCs) between September 1, 2023, and October 31, 2023. We recruited GAD patients from the Department of Psychiatry, Bangabandhu Sheikh Mujib Medical University Hospital, a tertiary-level teaching hospital in Dhaka city, Bangladesh, and HCs from surrounding areas of patients. A psychiatrist with expertise in neuropsychiatric disorders evaluated the subjects and diagnosed the GAD patients based on the Diagnostic and Statistical Manual for Mental Disorders, 5^th^ edition (DSM-5) criteria. Individuals having intense anxiety, fear, or worry about several everyday events or aspects for most of the days over an extended period of not less than six months were selected as GAD patients. The severity of GAD was assessed using the GAD-7 scale (total score ranges from 0 to 21)^46^. Patients having GAD-7 scores of higher or equal to five were selected for this study. Besides, patients having comorbidity with major depressive disorder, obsessive–compulsive disorder, panic disorder, post-traumatic stress disorder, social phobia, schizophrenia, Alzheimer’s, and Parkinson’s disease were excluded from the study. We also considered other exclusion criteria for the enrollment of patients. For example, patients having chronic infectious diseases, chronic liver and kidney diseases, and patients having a history of alcohol and substance use were excluded from the study population. Participants were limited to those who had not taken anxiolytics, antidepressants, or antipsychotic medications that could have affected serum IL-17A and IL-23A levels in the two weeks before the study. Patients who were exposed to glucocorticoids, immunomodulatory drugs or therapies, and anticancer drugs that may affect cytokine levels were also excluded from the study protocol. Pregnant patients or those with comorbidities from other AXIS I disorders were excluded. Individuals with cognitive disability, mutism, or non-participation were also not allowed in the study. Data on sociodemographic characteristics was acquired using a pre-designed questionnaire.

### Blood sample collection, processing, and storage

Five-milliliter blood samples were obtained from each study subject using established sampling methodologies. Following the collection process, the blood was left undisturbed in a Falcon tube for an hour to aid coagulation. The serum was then separated from the initial blood sample by centrifugation at 1000 *g* for 15 min at 25 °C. Following centrifugation, the separated serum was placed into an Eppendorf tube and stored at − 80 °C.

### Estimation of serum interleukin levels by ELISA assays

Serum levels of IL-17A and IL-23A were estimated by enzyme-linked immunosorbent assays using the human IL-17A ELISA kit PicoKine and the human IL-23 ELISA kit PicoKine (Boster Bio, USA), respectively, according to the manufacturer’s instructions. Briefly, 100 µl of sample and a standard cytokine solution was added to each well of the pre-coated 96-well microplate. The plate was then covered with a plate sealer and incubated for 120 min at room temperature. Subsequently, the liquid contents from each well were discarded, and 100 µl of biotinylated anti-IL-17A antibody or anti-IL-23A antibody were incorporated into each well of the microplate. After being covered with a sealer, the plate was subjected to incubation again at 37 °C for 60 min. The liquid contents of each well were then extracted, followed by washing the plate with 300 µl of wash buffer three times. After that, 100 µl of the avidin–biotin-peroxidase complex was added to each well and incubated at 25 °C for 40 min. Following the discarding of the liquid contents, the plate was washed five more times using 300 µl of wash buffer. 90 µl of color-developing reagent (TMB) was then incorporated into each well, and the plate was incubated in a dark place for 30 min. To stop the reaction process, 100 µl of stop solution was then added to the plate, and the OD absorbance at 450 nm for each well was determined by a microplate reader. Standard curves for both IL-17A and IL-23A were prepared, and concentrations of serum levels of specific cytokines for each sample were then measured in pg/ml by extrapolating the OD absorbance value into a standard equation derived from the respective standard curve. Serum cytokine measurements were performed in duplicate to ensure the robustness and reliability of the results.

### Statistical analysis

The data analysis was performed using GraphPad Prism v5.0b (GraphPad, San Diego, USA) and Statistical Package for the Social Sciences v24.0 (IBM Corp., Armonk, NY). Microsoft Excel 2019 was utilized to sort and organize the data. The demographic and clinical variables were described using descriptive statistics, with the results presented as the mean ± standard deviation (SD). The differences in demographic variables between patients and HCs were assessed using an unpaired, two-tailed Student’s t-test (for continuous variables) or Chi-square test (for categorical variables). The serum cytokine levels of patients with GAD and HCs were compared using a two-tailed, unpaired Student’s t-test. Dot-plot and scatter-plot graphs were used to visually compare the levels of cytokines between the HC and patient groups. Pearson’s correlation coefficient was used to analyze the association between cytokine levels and disease severity, as measured by GAD-7 scores. To differentiate between patients with GAD and HCs, we evaluated receiver operating characteristics (ROC) curves specifically for IL-17A and IL-23A levels. Statistical significance was considered at p < 0.05 in all instances.

### Ethical consideration

The research ethics committee of University of Asia Pacific approved this research protocol (UAP/REC/2023/202-S). The study’s aims were effectively conveyed to the participants, and each individual provided informed written consent. We conducted this inquiry in adherence to the guiding principles outlined in the Helsinki Declaration.

## Results

### Socio-demographic profiles of study population

A detailed analysis of the socio-demographic characteristics of the study population, drawing comparisons between GAD patients and HCs, is presented in Table 1. The mean age of GAD patients was 31.04 ± 1.52 years, while that of HCs was 30.66 ± 2.04 years, indicating no statistically significant difference (p = 0.878). An analysis of the age distribution among the following categories (18–25, 26–35, 36–45, and 46–60 years) unveiled proportional similarities between the two cohorts. The gender distribution of GAD patients (male: 60.00% and female: 40.00%) and HCs (male: 60.50% and female: 39.50%) was equal, with no difference between the sexes that was statistically significant (p > 0.999). An equivalent pattern was observed in the distribution of marital status: unmarried: 60.00%, HCs 68.42%; married: 40.00%, HCs 31.58%; no significant difference (p = 0.504). In comparison to HCs, GAD patients had a mean BMI of 24.70 ± 0.71 kg/m^2^, whereas HCs had a mean BMI of 23.49 ± 0.566 kg/m^2^; this difference was not statistically significant (p = 0.179). The proportions of individuals in the BMI categories (below 18.5, 18.5–25, and above 25) were also similar between the two cohorts. In terms of educational attainment, a significant percentage of both the patient population (40.00%) and HCs (44.74%) possessed a graduate degree or higher. A significant proportion of participants in both cohorts are classified as having a median economic status; specifically, 76.00% of patients and 84.21% of HCs are members of this group. Analysis of occupation, smoking history, and residence area did not reveal any statistically significant differences between GAD patients and HCs in these parameters (p > 0.05).

**Table 1:** Socio-demographic characteristics of the study population.

Parameters	GAD patients (n = 50) Mean ± SD	Healthy controls (n = 38) Mean ± SD	p value
Age in years	31.04 ± 10.80	30.66 ± 12.58	0.878
18–25	20 (40.00%)	14 (36.84)	
26–35	19 (38.00%)	16 (42.11)	
36–45	3 (6.00%)	2 (5.26)	
46–60	8 (16.00%)	6 (15.79)	
Sex			0.992
Male	30 (60.00%)	23 (60.50%)	
Female	20 (40.00%)	15 (39.50%)	
Marital status			0.504
Married	20 (40.00%)	12 (31.58%)	
Unmarried	30 (60.00%)	26 (68.42%)	
BMI (kg/m^2^)	23.49 ± 4.00	24.70 ± 4.37	0.179
Below 18.5 (CED)	3 (6.00%)	4 (10.53%)	
18.5–25 (normal)	31 (62.00%)	15 (39.47%)	
Above 25 (obese)	16 (32.00%)	19 (50.00%)	
Education level			0.244
Illiterate	4 (8.00%)	0 (0.00%)	
Primary level	7 (14.00%)	6 (15.79%)	
Secondary level	5 (10.00%)	1 (2.63%)	
Higher Secondary level	14 (28.00%)	14 (36.84%)	
Graduate and above	20 (40.00%)	17 (44.74%)	
Occupation			0.861
Housewife	12 (24.00%)	6 (15.79%)	
Business	5 (10.00%)	3 (7.89%)	
Unemployed/pensioner	14 (28.00%)	13 (34.21%)	
Student	12 (24.00%)	11 (28.95%)	
Others	7 (14.00%)	5 (13.16%)	
Economic status			0.477
High	2 (4.00%)	2 (5.26%)	
Medium	38 (76.00%)	32 (84.21%)	
Low	10 (20.00%)	4 (10.53%)	
Smoking history			0.131
Nonsmoker	43 (86.00%)	37 (97.37%)	
Smoker	7 (14.00%)	1 (2.63%)	
Residence area			0.830
Rural	21 (42.00%)	15 (39.47%)	
Urban	29 (58.00%)	23 (60.53%)	
Family history of GAD			**0.004**
Yes	10 (20.00%)	0 (0.00%)	
No	40 (80.00%)	38 (100.00%)	
Previous history of GAD			** < 0.001**
Yes	20 (40.00%)	0 (0.00%)	
No	30 (60.00%)	38 (100.00%)	

Significant values are in bold.

*BMI* body mass index, *CED* chronic energy deficiency, *GAD* generalized anxiety disorder, *SD* standard deviation.

Data were analyzed either by unpaired two tailed students t test when variables (age and BMI) are normally distributed or by Chi-square ( and Fisher’s exact test) for categorical data to determine the level of significance between mean difference between GAD patients vs control groups where p < 0.05 is considered to be statistically significant.

### Laboratory findings and clinical characteristics

**Figure 1:**
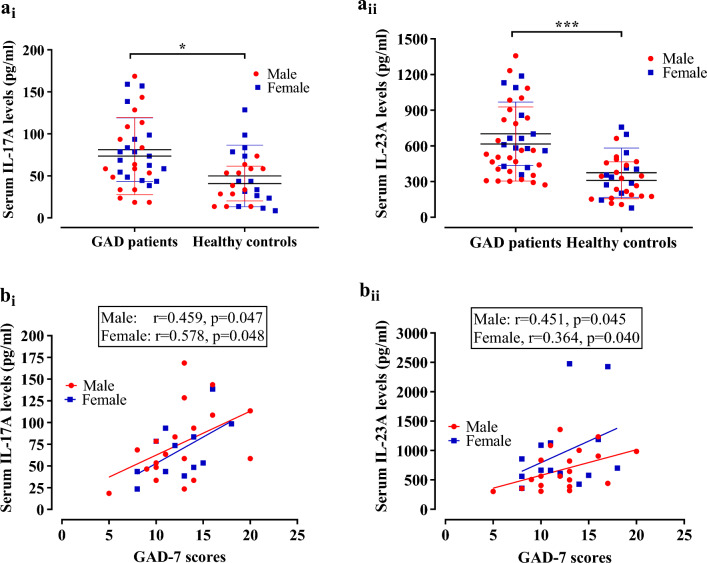
Comparison of serum IL-17A (a_i_) and IL-23A (a_ii_) levels between GAD patients and healthy controls. Data were presented as mean ± SD and analyzed by two tailed unpaired t test to determine the statistical level of significance between mean difference of two groups where p < 0.05 is considered to be statistically significant. [*] represents p < 0.05 and [***] represents p < 0.001. Scatter diagram of GAD-7 scores vs serum IL-17A levels (b_i_) and serum IL-23A levels (b_ii_) in the patient group were presented. Pearson correlation analysis was performed to find out the potential association between serum cytokine levels and GAD-7 scores of GAD patients. Both the male (red colored circular point) and female (blue colored rectangular point) GAD patients showed significant association between IL-17 or IL-23 serum levels and GAD-7 scores.

The clinical characteristics and laboratory findings are shown in Table 2. The mean DSM‐5 and GAD-7 scores for GAD patients were 9.62 ± 1.19 and 13.08 ± 4.02, respectively. Examining the interleukin levels, GAD patients displayed higher IL-17A (77.14 ± 58.30 pg/ml) compared to HCs (43.50 ± 25.54 pg/ml) (Fig. 1ai), indicating a statistically significant difference (p = 0.014). Further, stratification by sex revealed significantly elevated IL-17A levels in both male (73.50 ± 45.83 pg/ml) and female (81.00 ± 70.57 pg/ml) GAD patients compared to their respective counterparts in HCs (male: 40.81 ± 20.68 pg/ml, female: 50.72 ± 39.06 pg/ml) (p = 0.024 and p = 0.190, respectively). In terms of IL-23A, GAD patients exhibited substantially higher levels (644.90 ± 296.70 pg/ml) than HCs (334.40 ± 176.0 pg/ml) (Fig. 1aii), indicating a highly significant difference (p < 0.001). Further analysis by gender demonstrated elevated IL-23A levels in both male (615.60 ± 311.5 pg/ml) and female (701.50 ± 267.50 pg/ml) GAD patients compared to their respective counterparts in HCs (male: 310.40 ± 155.20 pg/ml, female: 374.30 ± 207.10 pg/ml) (p < 0.001 and p = 0.002, respectively) (Fig. 1 and Table 2). It is imperative to evaluate the gender-specific differences in cytokine levels in GAD patients. Though female GAD patients showed higher levels of IL-17A (81.00 ± 70.57 pg/ml) than their male counterparts (73.50 ± 45.83 pg/ml), the difference was not statistically significant (p = 0.718, two-tailed student t-test). Another important finding was that male (615.6 ± 311.50 pg/ml) and female (701.5 ± 267.50 pg/ml) GAD patients showed no significant variation in serum IL-23 levels (p = 0.385, two-tailed student t-test).

**Table 2:** Clinical characteristics and laboratory findings of the study population.

Parameters	GAD patients (n = 50) Mean ± SD	Healthy controls (n = 38) Mean ± SD	p value
DSM‐5 scores	9.62 ± 1.19	–	–
GAD-7 scores	13.08 ± 4.02	–	–
IL-17A (pg/ml)	77.14 ± 58.30	43.50 ± 25.54	**0.014**
Male	73.50 ± 45.83	40.81 ± 20.68	**0.024**
Female	81.00 ± 70.57	50.72 ± 39.06	0.190
IL-23A (pg/ml)	644.90 ± 296.7	334.40 ± 176.0	** < 0.001**
Male	615.60 ± 311.5	310.40 ± 155.2	** < 0.001**
Female	701.50 ± 267.5	374.30 ± 207.1	**0.002**

Significant values are in bold.

Two-tailed unpaired student’s *t* test was applied for evaluating statistical significance level between mean difference between cases and controls and p < 0.05 was statistically significant.

*DSM-5* diagnostic and statistical manual for mental disorders, 5th edition: *GAD* generalized anxiety disorder, *GAD-7* generalized anxiety disorder 7-item scores, *SD* standard deviation, *IL-17A* interleukin 17A, *IL-23A* interleukin 23A.

### Association between different demographic and clinical covariates with GAD severity

The correlation between IL-17A and GAD-7 scores was found to be statistically significant (r = 0.397, p = 0.032), with the correlation being most pronounced among females (r = 0.578, p = 0.048) and approaching significance among males (r = 0.459, p = 0.047) (Fig. 1bi). In a similar vein, there was a noteworthy correlation between IL-23A and GAD-7 scores (r = 0.359, p = 0.039), with significance being reached in males (r = 0.451, p = 0.045) and approaching significance in females (r = 0.364, p = 0.040) (Fig. 1bii). Significantly, a robust positive correlation (r = 0.533, p = 0.001) was identified between IL-23A and IL-17A, suggesting a strong association between these two cytokines (Table 3). Conversely, there was no statistically significant correlation observed between age and IL-17A (r = 0.169, p = 0.346) or IL-23A (r = -0.062, p = 0.698). Comparably, there was no significant correlation observed between BMI and the scores of IL-17A (r =− 0.098, p = 0.599), GAD-7 (r = 0.061, p = 0.675), or IL-23A (r = − 0.106, p = 0.521) (Table 3). We also performed Spearman correlation analysis between gender and IL-17A or IL-23A to evaluate the potential association between gender and tested interleukins. Analysis showed that there was no association between gender and IL-17A levels (Spearman r = 0.005, p = 0.976) or IL-23A levels (Spearman r = − 0.143, p = 0.348) (Table 3).

**Table 3:** Correlation between GAD-7 score and different demographic and clinical covariates.

Correlation parameters	Correlation analysis results	
	r	p
Age and IL-17A	0.169	0.346
Age and GAD-7 scores	0.043	0.763
Age and IL-23A	− 0.062	0.698
BMI and GAD-7 scores	0.061	0.675
BMI and IL-17A	− 0.098	0.599
BMI and IL-23A	− 0.106	0.521
IL-17A and GAD-7 scores	0.397	**0.032**
IL-23A and GAD-7 scores	0.359	**0.039**
IL-23A and IL-17A	0.533	**0.001**
*Gender and IL-17A	0.005	0.976
*Gender and IL-23A	− 0.143	0.348

Significant values are in bold.

Pearson’s correlation co-efficient, r represents level of association and p < 0.05 was considered to be statistically significant.

*BMI* body mass index, *GAD-7* generalized anxiety disorder 7-item scores, *IL-17A* interleukin 17A, *IL-23A* interleukin 23A.

*The correlation between gender and IL-17A or IL-23A were assessed by spearman correlation analysis where Spearman r represents Spearman’s correlation co-efficient and p < 0.05 is considered as statistically significant.

### Receiver operating characteristics analysis

**Figure 2:**
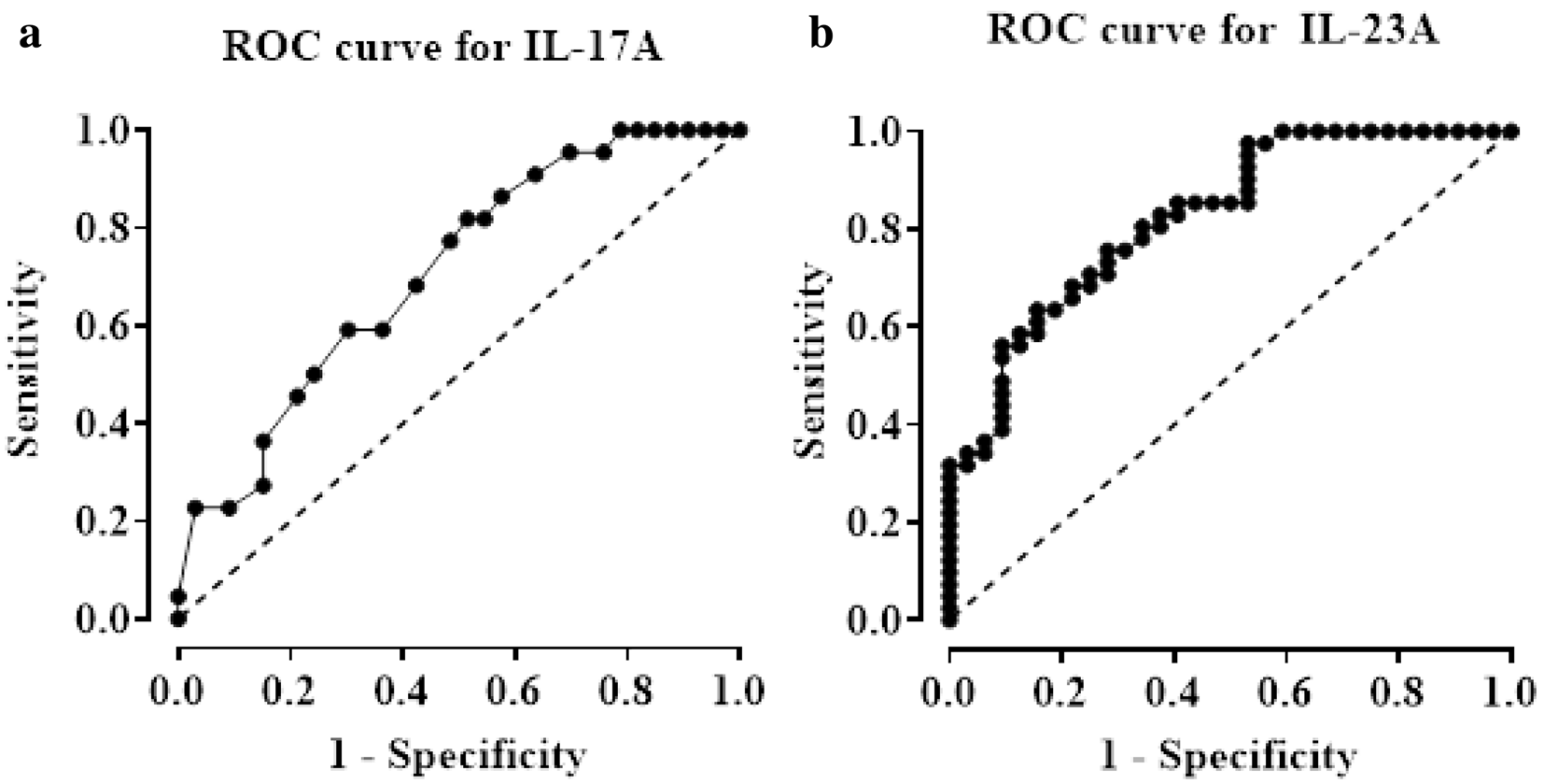
Receiver Operating Characteristic (ROC) curve for serum IL-17A levels (**a**) and IL-23A levels (**b**) in discriminating GAD patients from healthy controls.

We conducted a diagnostic accuracy evaluation using ROC analysis to investigate the capability of serum IL-17A and IL-23A concentrations to distinguish between persons with GAD and HCs. The investigation showed that the serum IL-17A levels exhibited a promising predictive value, with an Area Under the Curve (AUC) of 0.710 (95% CI: 0.572 to 0.844, p = 0.009). The sensitivity and specificity were determined to be 77.27% and 51.52%, respectively, employing a cut-off point value of 61.00 pg/ml. Compared to IL-17A levels, the levels of serum IL-23A demonstrated better performance in differentiating GAD patients from HCs, as indicated by a higher AUC value of 0.824 (95% CI: 0.731 to 0.918, p < 0.001). The sensitivity and specificity were determined to be 75.60% and 71.88%, respectively, using a cut-off value of 416.00 pg/ml (Table 4 and Fig. 2). Though both markers showed promising predictive values, the observed sensitivity and specificity values for both interleukins are lower that 80%, implicating that IL-17A and IL-23A serum levels failed to exhibit good accuracy in diagnosing GAD patients. These lower values for sensitivity and specificity data might be due to the smaller sample size and thus further studies using larger sample size is required to evaluate the diagnostic efficacy of these two markers.

**Table 4:** Receiver operating characteristic curve analysis of serum IL-17A and IL-23A levels as discriminators between GAD patients and healthy controls.

Cytokines	Cut-off values (pg/ml)	AUC	95% CI		Sensitivity (%)	Specificity (%)	p value
			Lower limit	Upper limit			
IL-17A	61.00	0.710	0.572	0.844	77.27	51.52	0.009
IL-23A	416.00	0.824	0.731	0.918	75.60	71.88	< 0.001

*AUC* area under the curve, *CI* confidence interval, *GAD* generalized anxiety disorder, *IL-17A* interleukin-17A, *IL-23A* interleukin-23A.

## Discussion

This study provides new insights into the correlation between serum levels of IL-17A and IL-23A and the severity of GAD in patients from Bangladesh. Our findings revealed that individuals with GAD exhibited heightened pro-inflammatory responses, as evidenced by significantly elevated serum levels of IL-17A and IL-23A compared to HCs. It supports the findings published by a limited number of studies^16,45^, which also revealed elevated levels of IL-17A in patients with GAD. Moreover, our research demonstrates a moderately strong and positive association between the increased concentrations of both IL-17A and IL-23A and the severity of GAD, as assessed by GAD-7 scores, by the utilization of Pearson correlation analysis. Our result is consistent with the results of another study^43^, which discovered a comparable relationship between levels of IL-23A and anxiety scores in individuals with GAD linked to inflammatory conditions such as psoriatic arthritis. Our inquiry is consistent with the findings of Liu and his colleagues^42^. They observed that IL-17 serum measurement was significantly and independently associated with anxiety severity in rheumatoid arthritis patients. Our findings indicate that peripheral levels of IL-17A and IL-23A may be associated with the pathophysiology and development of GAD. These results support the immune hypothesis of the development of GAD that a dysregulated immune system is associated with GAD pathophysiology.

The present study goes beyond analyzing cytokines due to the thorough evaluation of GAD patients. It uncovers increased scores according to the DSM-5 and the GAD-7, highlighting the seriousness of the disease. Distinct gender-specific differences arose, as both male and female patients with GAD displayed significant increases in levels of IL-17A and IL-23A compared to their counterparts in HCs. Our correlation study revealed moderately strong relationships, highlighting the interaction between IL-17A and GAD-7 scores, with variations distinct to each sex. The strong positive connection between IL-23A and IL-17A highlights the interdependence of these cytokines, revealing possible implications for the pathophysiology of GAD. In addition, our study examined demographic variables and found that age, sex, and BMI did not exhibit any significant associations with tested biomarkers. This element introduces intricacy to the complex link between demographic parameters and reported variations in cytokine levels in GAD, hence providing opportunities for additional exploration into the interaction of these variables.

The pathophysiological mechanisms of GAD are extensive and involve complex interplay between neurological, genetic, and environmental variables^47^. The correlation between blood levels of IL-17A and IL-23A and the severity of GAD, as demonstrated in our work, provides an essential understanding of the probable immunological pathways. An important aspect of GAD pathogenesis is immune system disruption^48^. Our research emphasizes the significance of pro-inflammatory cytokines (IL-17A and IL-23A) in this mechanism. IL-17A, predominantly secreted by T-helper 17 (Th17) cells, is a very influential pro-inflammatory cytokine recognized in numerous autoimmune and inflammatory disorders^18,49^. IL-23A has a vital role in the differentiation and upkeep of Th17 cells. The positive correlation between serum IL-17A and IL-23A levels in GAD patients explains that IL-17 secretion from macrophages or other immune cells is due to the stimulation by IL-23^50^. This interaction can enhance the inflammatory process in GAD patients. The consequences of this interconnection go beyond linear connections, highlighting the need to investigate the dynamic interactions among these cytokines in the context of GAD pathogenesis.

Our study highlights the potential of measuring IL-23A serum levels as a probable diagnostic biomarker for GAD. Incorporating IL-23A into diagnostic methods may improve precision and facilitate the early assessment of GAD risk. The higher AUC value (0.824) exhibited in ROC analysis with commendable sensitivity (75.60%) at a specified cut-off value indicates that IL-23A may distinguish GAD sufferers from HCs. However, the lower level of sensitivity (71.88%) displayed by the serum IL-23A measurement implies that IL-23A failed to exhibit good diagnostic performance and urges further studies with a larger population size to evaluate the exact role of IL-23A in GAD pathophysiology. Although IL-17A initially displayed a moderate connection with GAD-7 scores in the study that considered one variable at a time, it performed poorly in accurately distinguishing between GAD patients and HCs. It was evident from the low specificity and low sensitivity seen in the ROC analysis. However, these lower levels of sensitivity or specificity might be due to the lower sample size of our study. These results demonstrated that serum IL-17A levels might not be an effective risk predictor for GAD development or further case–control studies using larger population sizes or longitudinal studies are needed to evaluate its accurate diagnostic efficacy. It highlights the significance of prudence when depending exclusively on IL-17A for diagnostic purposes. Additional studies, which may include larger groups of participants and studies conducted over a long period, are necessary to clarify its diagnostic capabilities and enhance its position in the diagnostic field of GAD.

Another implication of our study findings is that our results potentiate the idea that the IL-23A and IL-17A immune axis might be exploited the development of novel anxiolytic/antidepressant or anti-inflammatory drugs targeting soluble IL-17A/ IL-23A cytokine protein or IL-17A receptor/ IL-23A receptor-mediated signaling pathway^51^. Currently, some anti-IL-17A (secukinumab, ixekizumab, bimekizumab, brodalumab, etc.) and anti-IL-23A (ustekinumab) monoclonal antibodies are used for the treatment of different autoimmune disorders including IBD, ulcerative colitis, psoriasis arthritis, rheumatoid arthritis, etc.^52,53^. Our observation that the IL-23A/ IL-17A immune axis is associated with anxiety severity suggested that either these immunological factors are acting as the causative agents for GAD development by inducing excessive neuroinflammation in critical brain regions associated with fear or anxiety processing or higher levels of these Th-17 cytokines are just a reflection of the pathogenesis of anxiety disorder. In both cases, these two cytokines may associate with the pathophysiology of anxiety disorder. Thus, we can target them for anxiolytic and or anti-inflammatory drug development. A clinical trial supports our study finding where administration of ustekinumab, an anti-IL-23A monoclonal antibody, or brodalumab, or anti-IL-17A blockers resulted in a significant reduction of anxiety and depression symptoms in psoriasis patients^54,55^. The discovered connection between increased levels of IL-17A and IL-23A and the intensity of anxiety in patients with GAD provides opportunities for therapeutic intervention. Investigating immunomodulatory strategies targeting IL-17A and IL-23A pathways may offer promising therapeutic options for GAD. Anti-cytokine treatments, known for their effectiveness in treating several autoimmune and inflammatory disorders^52,53^, should be explored for their ability to reduce the intensity of pro-inflammatory reactions in GAD. However, understanding the immune system’s delicate balance requires careful consideration, and developing targeted treatments relies on a thorough understanding of the specific processes involved in GAD development. Our data highlights the significance of tailoring treatment approaches to individual patients, considering the differences in IL-17A and IL-23A levels between males and females. Customizing therapies according to patient profiles encompasses immunological biomarkers and gender-related characteristics that may optimize therapy efficacy and results. Our findings have practical significance for public health and can be used to develop targeted preventative initiatives. Integrating the finding of IL-23A as a promising diagnostic biomarker into screening programs for groups at higher risk for anxiety disorders could be beneficial. Regular surveillance of IL-23A levels, particularly in those with a familial background or other predisposing factors for GAD, might facilitate early detection and prompt therapies, potentially halting the development of clinically significant anxiety.

### Strength and limitation of this study

The strength of our study is that we have applied a range of analyses to explore the potential association between serum levels of IL-23 and IL-17 and anxiety severity and to evaluate the potential for these two cytokines to act as risk predictors of developing GAD. As per our knowledge, this is the first study in which we have observed a moderately strong but significant positive correlation between IL-23A levels and GAD. The strict design of the study protocol with a set of inclusion and exclusion criteria helps us to generate a more or less homogenous population of the Bangladeshi cohort. Besides, the selection of age, sex, and BMI-matched HCs enabled a robust comparison of cytokine serum levels between patients versus control groups. Another strength of our study is that we considered a range of confounding variables and thereby tried to minimize the effect of confounding variables such as age, sex, BMI, co-morbid diseases, and immunomodulatory drugs on cytokine serum levels.

Despite having some strengths, our study has some limitations that we should acknowledge. Though peripheral IL-17 and IL-23 levels are associated with anxiety severity in our study, how this IL-23/ IL-17 immune axis mediates such anxiety symptoms in GAD patients remains unexplored or unclear. Another limitation is that we cannot provide a definite conclusion regarding the causal relationship between elevated serum levels of cytokines and anxiety states due to the nature of the study design. A case–control study is not enough to establish a causal relationship between variables. Further research including longitudinal studies are warranted to establish the causal relationship between disease severity and Th17 cytokine-mediated inflammatory responses. Another limitation of our study is that we employed only 50 GAD patients and 38 HCs. This small sample size is a limitation as a small sample size cannot truly represent the whole population of Bangladesh due to the high background noise in statistical analysis.

### Future study direction

Increasing the size of the study groups could improve the applicability of our results and offer a more thorough comprehension of the connection between cytokines and GAD. This study predominantly depends on serum cytokine measures, providing a viewpoint from the periphery. Analyzing the CSF fluid or using neuroimaging techniques to investigate the neuroinflammatory elements of GAD are required to understand the involvement of the central nervous system. In addition, although our study accounted for specific confounding factors, we did not thoroughly consider other potential variables that could affect cytokine levels, such as environmental factors, lifestyle factors, diet, or genetic factors such as genetic polymorphisms in cytokine genes. Future research should incorporate a broader range of variables to explore the actual understanding of GAD pathophysiology.

## Conclusion

Our study provides valuable insights regarding the association between serum IL-17A and IL-23A levels and the pathophysiology of GAD. The current research supports the immunological theory of GAD pathophysiology by highlighting the possible roles of IL-23 and IL-17 in the immune axis. These findings may guide the identification of novel blood-based biomarkers for GAD. It can also help to develop novel anxiolytic drugs targeting the IL-23/IL-17 immune axis. To the best of our knowledge, we, for the first time, evaluated the potential for IL-17A and IL-23A serum levels as diagnostic biomarkers for GAD among the population in Bangladesh. Our research contributes to the dynamic field of GAD, laying the groundwork for further investigation and improvement of diagnostic and therapeutic approaches. Based on the present study, we recommend further research using a larger population size to investigate the accurate diagnostic efficacy of these biomarkers.

## Data Availability

The datasets used and/or analyzed during the current study available from the corresponding author on reasonable request.
